# Date of birth and purchase price as foals or yearlings are associated with Thoroughbred flat race performance in the United Kingdom and Ireland

**DOI:** 10.1002/vro2.43

**Published:** 2022-09-23

**Authors:** Juan Carlos Arango‐Sabogal, Rebecca Mouncey, Amanda M. de Mestre, Kristien Verheyen

**Affiliations:** ^1^ Department of Pathobiology and Population Sciences The Royal Veterinary College Hatfield Hertfordshire UK; ^2^ Département de Pathologie et Microbiologie, Faculté de Médecine Vétérinaire Université de Montréal Saint‐Hyacinthe Québec Canada; ^3^ Department of Comparative Biomedical Sciences The Royal Veterinary College Hatfield Hertfordshire UK

## Abstract

**Background:**

Thoroughbred breeders aim to have foals born early in the season, but scientific evidence on the advantages for race performance is scarce and contradictory.

**Methods:**

The association between date of birth and purchase price as foal/yearling, with race performance by the end of the second and third years of life of Thoroughbreds racing in flat races in the United Kingdom (UK) and Ireland (IRE) was assessed using negative binomial and zero‐inflated negative binomial models on the entire 2014–2015 UK/IRE foal crops (*n* = 28,282).

**Results:**

In total, 6666 and 9456 horses raced in UK/IRE flat racing by the end of their second and third years of life. Prize money and prize money per start decreased with each additional day beyond 1 January that the foal was born. Purchase price as foal and yearling was negatively associated with the number of races run, while it was positively associated with prize money and prize money per start by the end of the third year of life.

**Conclusions:**

Foals born early in the season had higher earnings by the end of their second and third years of life than foals born later. Differences were more marked among males than females. The most expensive horses sold as foals or yearlings ran fewer races but earned more prize money and prize money per start than less expensive horses. Results from this population‐based analyses may inform strategies and management practices aiming to maximise horses’ racing performance potential and increase financial returns.

## INTRODUCTION

The cost of producing a Thoroughbred (TB) racehorse from birth to entering training at around 18 months of age has been estimated to be £30,000–£42,000 (Tattersalls Book 3 and Book 1, respectively; www.tattersalls.com/sales) excluding stallion covering fees.^1^ Returns on such investments are only achieved either by sale or from prize money won during an individual's racing career.^1^ It is estimated that almost 60% of breeders breed TBs both for sales and racing,^1^ making it vital that individuals produced are capable of achieving maximal financial return at weanling and/or yearling sales^2^ and/or reach their peak athletic performance at 2 and 3 years of age, when the majority of opportunities to earn prize money occur in the flat racing calendar.^3^ In the Northern Hemisphere, the official date of birth (DoB) of TBs is 1 January of the corresponding year of birth. Current industry convention is to maximise efforts to produce foals as early in the breeding season as possible, given the likelihood of such individuals to be physically precocious, which may be advantageous both in the sales ring and on the racecourse.

Evidence supporting an association between DoB and race performance is scarce and contradictory. In an United Kingdom (UK) study conducted on 1022 TBs, horses born between January and March were more likely to race as 2‐year‐old horses compared to horses born later in the season; birthdate made no difference to their likelihood of winning.^3^ Two French studies concluded that horses born early in the season performed better than horses born later.^4^, ^5^ In Finland, Standardbred and Finnhorse trotters born early in the season were faster, had greater earnings and larger number of placings than horses born late in the season.^6^ Other studies have not reported an association between DoB and racing performance milestones or outcomes or,^7^, ^8^, ^9^ conversely, reported that earlier born foals had a shorter racing career than those born later.^10^

Purchase price as yearling has been associated with race performance of 2‐ and 3‐year‐old horses in Australia^11^ and with the end‐of‐year Timeform rating at 3 years in the UK and Ireland (IRE).^12^ However, these studies analysed data on yearling sales only, using a cohort of 2773^11^ and 1735 horses,^12^ respectively. To the best of the authors' knowledge, no studies have analysed the associations between purchase price as foal and yearling with race performance using an entire foal crop population.

Our main objective was to assess the association between the horses’ DoB and their race performance by the end of their second and third years of life in UK/IRE flat racing. A secondary objective was to determine if purchase price as foal and yearling was associated with race performance by the end of their third year of life.

## MATERIALS AND METHODS

### Study design and study population

Data for this retrospective cohort study were provided under non‐disclosure agreements with both Weatherbys (www.weatherbys.co.uk) and the British Horseracing Authority. The source population, described elsewhere,^13^ included the entire 2014–2015 UK and IRE Thoroughbred foal crops (*n* = 28,282). Given that the outcomes for this study were measures of race performance by the end of horses’ second and third years of life in UK/IRE flat racing, the study population included all the horses that raced at least once in UK/IRE flat races by the end of their third year of life (*n* = 9456).

### Statistical analyses

**FIGURE 1:**
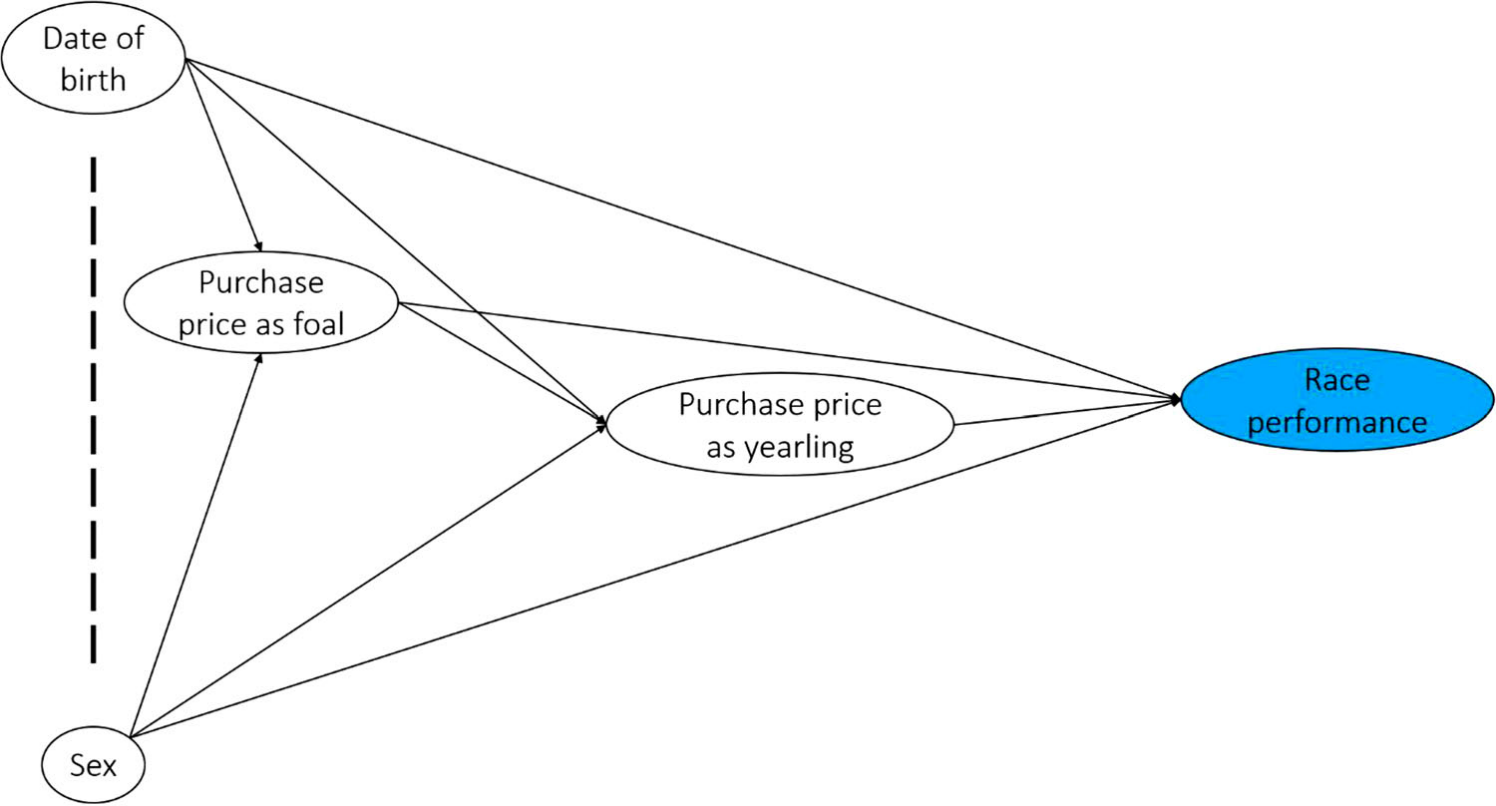
Theoretical directed acyclic graph illustrating the association of date of birth and purchase price, and race performance of 2014–2015 UK/Ireland Thoroughbred foal crops by the end of their second and third years of life. The dashed line connects exposures thought to be causally unrelated to each other.

The unit of interest was the horse. A directed acyclic graph (DAG) was built to estimate the total effects of the exposures of interest on the outcomes (Figure 1). Initially, the distributions of explanatory variables and the outcomes were described using median, mean, range, interquartile range (IQR) and standard deviation for continuous variables, and the frequency and proportions with 95% confidence intervals (CIs) for categorical variables.

#### Outcomes

Available race performance data for the study population comprised number of races, wins, places and prize money won from 2016 to 2018.^13^ Outcomes for the study included number of races, prize money and prize money earned per start in UK/IRE flat racing by the end of their second and third years of life, respectively.^14^ All the outcomes followed a negative binomial (NB) distribution (Table 1). Therefore, they were modelled as count data assuming that prize money and prize money per start were counts of £1 coin and £1 coin/start, respectively. The variable number of races was analysed using a NB regression model, whereas prize money and prize money per start were modelled using a zero‐inflated negative binomial (ZINB) model due to the high proportion of zeros observed in these two variables. The choice of model was confirmed during the multivariable analysis by running Poisson regression models and comparing them to their respective NB or ZINB models using the Akaike's and Bayesian information criteria.

**TABLE 1:** Descriptive statistics of performance outcomes of the 2014–2015 UK/Ireland (IRE) Thoroughbred foal crops in UK/IRE flat races by the end of their second and third years of life.

Age	Outcome	Median	Mean (standard error of mean)	Standard deviation	Interquartile range	Range
End of second year (*n* = 6666)	Number of races	3	3.6 (0.03)	2.3	2–5	1–17
	Prize money^a^	818	4889 (207.9)	16,974	0–4390	0–559,193
	Prize money^a^ per start	243	1183 (42.3)	3452	0–1143	0–93,198
End of third year (*n* = 9456)	Number of races	6	7.1 (0.05)	4.6	4–10	1–32
	Prize money^a^	3513	11,941 (478.6)	46,536	385–10,334	0–1,367,500
	Prize money^a^ per start	489	1545 (61.4)	5971	80–1356	0–214,945

^a^ Prize money in GBP (£, British pound sterling).

#### Explanatory variables

The minimal sufficient adjustment sets of predictors for each objective were identified based on the DAG (Figure 1). For the first objective, the exposure of interest was the horse's DoB, measured as the number of days from 1 January of the corresponding year of birth. This variable was scaled to reflect the changes for every 7 days after 1 January. The binary variable sex of the horse (male or female) was another explanatory variable considered in the model for the first objective. For the second objective, two exposures of interest were considered individually: purchase price as foal and purchase price as yearling. These variables included all horses that were brought to the sales event with the intention of being sold. Horses not sold during the sales event were classed as ‘went but not sold’. Horses sold at the sales event were grouped into four classes according to quartiles of the purchase price in Guineas paid for the transaction (1 Guinea = £1.05). When purchase price as yearling was the exposure of interest, purchase price as foal was explored as a confounder variable, adding a category ‘not sold as foal’ to include horses intended to be sold as a yearling but not intended to be sold as a foal. For horses with multiple sales transactions as yearlings, the average purchase price was calculated. The DoB and sex were considered in the models for the second objective.

#### Multivariable analyses

Selection of potential explanatory variables to be included in the multivariable models was based solely on the minimal sufficient adjustment sets for estimating the total effect of the exposures of interest and the outcomes,^15^, ^16^ according to the DAG (Figure 1). For the first objective, a model including DoB as the only predictor was proposed. This model was run for each outcome, resulting in a total of six models. In all models, two‐way interactions between DoB and sex were explored by comparing a model with interaction terms between these two variables to a model without interaction using the likelihood ratio test (LRT). When an interaction was observed (LRT, *p* < 0.05), the interaction term was retained in the models and results presented graphically. For the second objective, when purchase price as foal was the exposure of interest, the minimal sufficient adjustment set included DoB and sex as potential confounders (Figure 1). When purchase price as yearling was the exposure of interest, the minimal sufficient adjustment set included purchase price as foal, DoB and sex as potential confounders (Figure 1). Proposed models for the second objective were run for each performance outcome by the end of the third year of life, resulting in a total of six models.

When an association between the exposure of interest and the outcomes was observed, the predicted probabilities from the model were estimated and presented graphically, using the margins command in Stata. Statistical significance threshold was set at *p* <0.05. All analyses were conducted in Stata (Release 15. Stata Corp., College Station, TX, USA).

## RESULTS

### Study population and descriptive results

**FIGURE 2:**
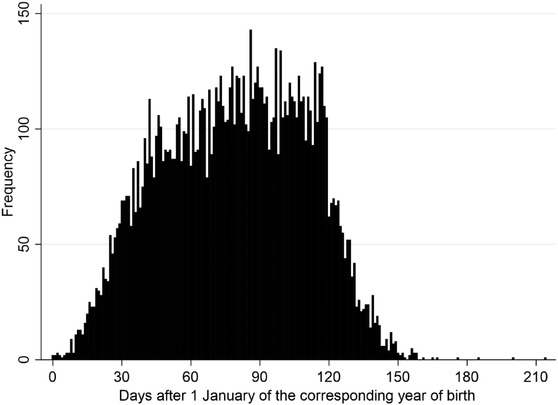
Distribution of date of birth measured as days after 1 January of the corresponding year of birth of 9456 UK/Ireland (IRE) Thoroughbred horses from the 2014–2015 foal crops that raced at least once in UK/IRE flat racing by the end of their third year of life.

Of the 2014–2015 foal crops (*n* = 28,282), 6666 and 9456 horses raced in UK/IRE flat racing by the end of their second and third years of life, respectively (Table 1). The number of races by the end of the second year of life varied from 1 to 17 (median = 3; IQR = 2–5) and by the end of the third year of life from 1 to 32 (median = 6; IQR = 4–10). Overall, 34% (*n* = 2271) and 19% (*n* = 1833) of the horses did not earn any prize money by the end of their second and third years of life, respectively. The distribution of the number of races, prize money earned and prize money per start is presented in Table 1. Horses were born between 0 and 214 days after 1 January of the corresponding year of birth, with 50% of the horses being born within 80 days after 1 January (Figure 2). There were more males (*n* = 4986; 52.7%; 95% CI: 51.7–53.7) than females (*n* = 4470; 47.3%; 95% CI: 46.3–48.3) in the study population.

In total, 6686 horses were intended to be sold and brought to a sales event, either as foals (*n*  = 2787) or yearlings (*n*  =  6288; Table S1). Of those intended to be sold as foals, 499 horses were brought to the sales event but were not sold. One sale transaction per individual was observed for the remaining horses sold as foals (*n* = 2288) and purchase price varied from 738 to 1,428,571 Guineas. Of the horses intended to be sold as yearlings, 547 were brought to the sales but were not sold. Purchase price of those horses sold as yearlings (*n* = 5741) varied from 560 to 2,600,000 Guineas. Most of these horses had one sale transaction per individual but some had two (*n* = 159) or three (*n* = 2) sale transactions as yearlings. The remaining 2770 horses were not sold either as a foal or yearling. The distribution of the categorical variables purchase price as foal and yearling is presented in Table S1.

Median prize money earned by the end of the third year of life was £3825 (IQR = 481–11,141; range: 0–864,285) for horses brought to a sales event as foals, £3768 (IQR = 481–10,541; range: 0–864,285) for those brought as yearlings and £3072 (IQR = 241–9909; range: 0–1,367,500) for those never brought to a sales event as foals or yearlings.

Only 17% (*n* = 387) of the horses sold as a foal and 13% (*n* = 746) of those sold as a yearling earned enough prize money by the end of their third year of life to cover their purchase price. On average, these horses earned £23,349 (median = 8805; IQR = 3285–21,334; range: 16–799,317) and £21,011 (median = 7338; IQR = 2599–18,303; range: 1–799,285) above the purchase price paid as foals and yearlings, respectively. For those horses earning less prize money than the purchase price paid, the purchase price as foal was on average £22,550 (median = 11,073; IQR = 4798–23,597; range: 9–1,425,561) higher than the prize money earned by the third year of life. The purchase price as a yearling was on average £47,840 (median = 17,500; IQR = 6301–46,248; range: 6–2,586,896) higher than the prize money earned by the third year of life.

Descriptive statistics of performance outcomes for horses not sold either as a foal or yearling (*n* = 2770) are presented in Table S2.

### Multivariable analyses results

#### Date of birth and race performance as 2‐ and 3‐year olds

No association was observed between DoB and the number of races by the end of the second (Coefficient = 0.001; 95% CI: −0.003, 0.004; *p* = 0.61) or third year of life (Coefficient =  −0.002; 95% CI: −0.005, 0.001; *p* = 0.19). Total prize money and prize money earned per start by the end of the second year of life decreased by 3% (both *p* < 0.001) for every 7 days after 1 January of the corresponding year of birth, while the odds of not earning any prize money increased by 3% (both *p* < 0.001; Table 2). No interaction was observed between DoB and sex for either total prize money or prize money per start by the end of the second year of life (*χ* ^2^ = 0.13; df = 1; *p* = 0.72 and *χ* ^2^ = 0.0; df = 1; *p* = 0.96, respectively).

**TABLE 2:** Final zero‐inflated negative binomial models to assess the association between date of birth of the 2014–2015 UK/Ireland (IRE) Thoroughbred foal crops and prize money, and prize money earned per start by the end of the second and third years of life in UK/IRE flat racing.

	*β* ^a^	95% Confidence interval	Exp (*β*)	*z*	*p*
**Prize money**					
**Second year of life (*n* = 6666)**					
Count part (negative binomial)					
Date of birth^b^	−0.03	−0.04; –0.02	0.97	−6.5	<0.001
Binary part (logistic)					
Date of birth^b^	0.03	0.02; 0.04	1.03	5.1	<0.001
**Third year of life (*n* = 9456)**					
Count part (negative binomial)					
Date of birth^b^	−0.03	−0.04; –0.02	0.98	−5.1	<0.001
Sex					
Female	Ref.	–	–	–	–
Male	0.42	0.26; 0.58	1.5	5.2	<0.001
Date of birth^b^ × sex	−0.02	−0.03; –0.003	0.98	−2.4	0.015
Binary part (logistic)					
Date of birth^b^	0.03	0.02; 0.05	1.03	4.1	<0.001
**Prize money per start**					
**Second year of life (*n* = 6666)**					
Count part (negative binomial)					
Date of birth^b^	−0.03	−0.04; –0.02	0.97	−6.7	<0.001
Binary part (logistic)					
Date of birth^b^	0.03	0.02; 0.04	1.03	5.1	<0.001
**Third year of life (*n* = 9456)**					
Count part (negative binomial)					
Date of birth^b^	−0.02	−0.03; –0.01	0.98	−5.2	<0.001
Sex					
Female	Ref.	–	–	–	–
Male	0.35	0.21; 0.51	1.4	4.6	<0.001
Date of birth^b^ × sex	−0.02	−0.03; –0.003	0.98	−2.4	0.017
Binary part (logistic)					
Date of birth^b^	0.03	0.02; 0.05	1.03	4.0	<0.001

^a^ *β*: regression coefficient.

^b^ Measured as days after 1 January of the corresponding year of birth and scaled to reflect the increase by every 7 days.

**FIGURE 3:**
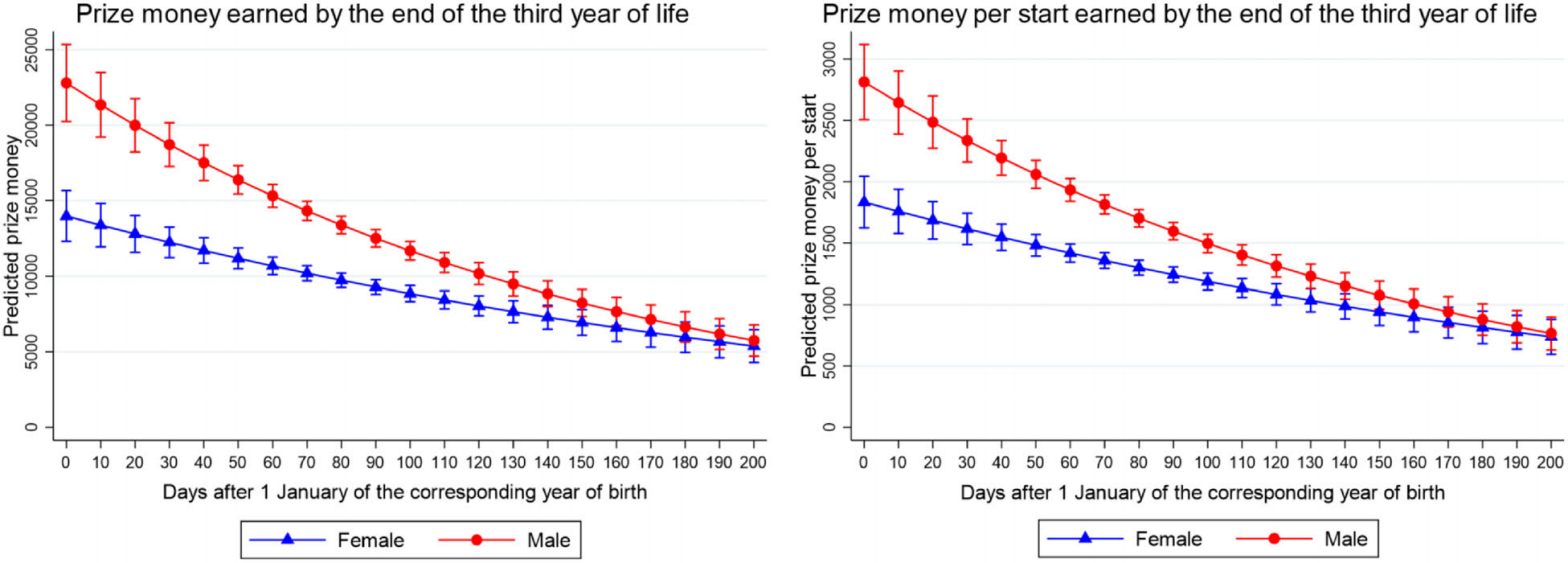
Predicted prize money (left) and prize money per start (right) earned by the 2014–2015 UK/Ireland (IRE) Thoroughbred foal crops by the end of their third year of life in UK/IRE flat racing. Marginal predictions and 95% confidence intervals were obtained using a zero‐inflated negative binomial regression to assess the association between horse's date of birth and prize money and prize money per start after controlling for the sex of the horses. Prize money in GBP (£)

In general, total prize money and prize money earned per start by the end of the third year of life decreased by 2% (both *p* < 0.001) for every 7 days after 1 January of the corresponding year of birth (Table 2). However, the interaction observed between DoB and sex for both these outcomes indicates that this association was different for males and females (Figure 3). No interaction between DoB and sex was observed for the binary part of the models. The overall odds of not earning prize money by the end of the third year of life increased by 3% for every 7 days after 1 January of the corresponding year of birth (both *p* < 0.001; Table 2).

#### Purchase price as foal and yearling, and race performance as 3‐year‐olds

The final models to assess the associations between purchase price as foal and yearlings, and the number of races run, prize money and prize money earned per start by the end of the third year of life in UK/IRE flat racing are presented in Tables S3–S5. The predicted number of races, prize money and prize money per start by the end of the third year of life (and 95% CI) by category of purchase price as a foal and yearling are presented in Table 3. The predicted probability (and 95% CI) of not earning any prize money and prize money per start by the end of the third year of life by category of purchase price as a foal and yearling is presented in Table 4.

**TABLE 3:** Predicted number of races, prize money, and prize money per start earned by the end of the third year of life of the 2014–2015 Thoroughbred foal crops in UK/Ireland flat races.

	Predicted outcomes and 95% confidence intervals by the end of the third year of life		
Purchase price category (in Guineas)	Number of races^a^	Prize money (£)^b^	Prize money (£) per start^b^
**As foal** ^**c**^ **(*n* = 6666)**			
Went to sale/not sold	7.3 (6.9–7.7)	6751 (5853–7649)	783 (683–882)
≤5167	8.2 (7.7–8.9)	8355 (6770–9940)	834 (683–984)
>5167–11,904	8.3 (7.8–8.8)	8535 (7306–9763)	912 (787–1037)
>11,904–25,396	8.1 (7.7–8.5)	10,234 (9085–11,382)	1239 (1106–1371)
>25,396	7.3 (7.0–7.6)	12,676 (11,548–13,768)	1692 (1553–1831)
**As yearling** ^**d**^ **(*n* = 9456)**			
Went to sale/not sold	6.7 (6.4–7.1)	8108 (7051–9165)	1038 (911–1116)
≤6666	7.8 (7.4–8.1)	5241 (4683–5798)	588 (529–648)
>6666–19,000	8.0 (7.7–8.3)	7512 (6899–8125)	849 (783–914)
>19,000–48,000	7.9 (7.7–8.2)	10,683 (9957–11,408)	1246 (1167–1326)
>48,000	6.9 (6.6–7.1)	15,462 (14,405–16,520)	2065 (1933–2197)

*Note*: Marginal predictions and 95% confidence intervals were obtained from negative binomial and zero‐inflated negative binomial models to assess the association between purchase price as foal or as yearling and racing performance outcomes.

^a^ Marginal predictions and 95% confidence intervals were obtained from a negative binomial model.

^b^ Marginal predictions and 95% confidence intervals were obtained from a zero‐inflated negative binomial model.

^c^ Estimates adjusted by date of birth and sex of the horses.

^d^ Estimates adjusted by date of birth, sex and purchase price as foal.

**TABLE 4:** Predicted probability of not earning prize money and prize money per start by the end of the third year of life of the 2014–2015 Thoroughbred foal crops in UK/Ireland flat races.

	Predicted probability (%) and 95% confidence interval	
Purchase price category (in Guineas)	Not earning prize money (£)^a^	Not earning prize money (£) per start^a^
**As foal** ^**b**^ **(*n* = 6666)**		
Went to sale/not sold	24.5 (24.2–24.7)	24.2 (24.0–24.4)
≤5167	28.9 (28.7–29.2)	28.7 (28.4–28.9)
>5167–11,904	24.0 (23.8–24.2)	23.8 (23.6–24.0)
>11,904–25,396	15.9 (15.8–16.1)	15.7 (15.6–15.9)
>25,396	11.8 (11.7–11.9)	11.7 (11.5–11.8)
**As yearling** ^**c**^ **(*n* = 9456)**		
Went to sale/not sold	25.1 (24.9–25.3)	24.9 (24.8–25.1)
≤6666	28.9 (28.7–29.2)	28.5 (28.4–28.7)
>6666–19,000	18.9 (18.8–19.0)	18.6 (18.5–18.7)
>19,000–48,000	15.3 (15.2–15.4)	15.1 (15.0–15.2)
>48,000	10.7 (10.6–10.8)	10.6 (10.5–10.6)

*Note*: Marginal predictions and 95% confidence intervals were obtained from the binary part of zero‐inflated negative binomial models to assess the association between purchase price as foal or as yearling and racing performance outcomes.

^a^ Marginal predictions and 95% confidence intervals were obtained from a zero‐inflated negative binomial model.

^b^ Estimates adjusted by date of birth and sex of the horses.

^c^ Estimates adjusted by date of birth, sex and purchase price as foal.

#### Purchase price as foal and yearling, and number of races

The predicted number of races by the end of the third year of life was lower for the most expensive foals and yearlings compared to horses in the other purchase categories. However, the predicted number of races by the end of the third year of life of the most expensive foals and yearlings was not different from that of horses brought to the sales event as foals or yearlings but not sold (Table 3).

#### Purchase price and prize money and prize money per start earned

Overall, the count part of the models suggested that prize money (Table S4) and prize money per start (Table S5) earned by the end of the third year of life increased with the purchase price as foal or yearling. The binary part of the models suggested that the odds of not earning any money (Table S4) and money per start (Table S5) by the end of the third year of life decreased as the purchase price as foal or yearling increased. The most expensive foals and yearlings earned on average more prize money and money per start by the end of the third year of life than horses in the other purchase categories (Table 3). The most expensive foals and yearlings had a lower predicted probability of not earning any prize money and prize money per start by the end of the third year of life, than horses in the other purchase categories (Table 4).

## DISCUSSION

To the best of the authors’ knowledge, this is the first UK study utilising population level that suggests that TB foals born early in the breeding season have better flat race performance by the end of the second and third years of life than later‐born foals. ‘Early’ foals earned more prize money and prize money per start than late foals, which was not related to the number of races run. Other studies analysing data from TBs,^4^, ^5^ and Standardbred and Finnhorse trotters,^6^ also reported higher earnings of early foals compared to late foals. Consistent with a previous study,^9^ we did not observe a difference in the number of races run by the end of the second and third years of life between earlier and later‐born foals.

The differences in race performance between early and late foals observed in our study are likely explained by variations in the growth rate related to real age differences among horses within the same administrative age category. Month of birth influences both bodyweight and average daily weight gain at different ages,^17^ with consistent differences through the first 2 years of life seen between foals born early and late in the year.^17^, ^18^ Growth rate in TBs accelerates from the first to the third months and from the 10th to the 16th months of life.^19^, ^20^ Developmental differences could be exacerbated if these periods of growth acceleration coincide with months in which temperature and pasture availability are favourable for both the mare and foal, influencing their average daily weight gain,^17^ as would be the case for foals born January–March, in which these growth periods coincide with spring pasture flush (March–May).

A previous study suggested that growth development was different between male and female TBs due to, among other factors, sex differences in energy balance mechanisms.^21^ It could be expected that the relationship between DoB and prize money and prize money per start would be different for males and females. Our results support such hypotheses, with males having overall higher earnings than females, and the reduction in prize money and prize money earned per start as DoB ‘increased’ since 1 January being more pronounced for males compared to females. A previous study evaluating the association between month of birth and race performance in different race types, including flat races, also found an interaction between DoB and sex.^5^ An alternative hypothesis is that relative opportunities to earn prize money may be different between colts and fillies. In 2020, the industry launched a bonus scheme where British‐bred fillies had the opportunity to earn £20,000 extra prize money in around 3000 eligible races.^22^ However, this scheme was not available to fillies during the present study. Considering such observations, it is important that future studies assessing race performance consider the real age (instead of the administrative age), the sex of the horses, relative numbers of eligible races, along with any such prize money bonus schemes in their analyses, as potential confounding factors or effect modifiers. Breeding strategies aiming to produce foals early in the season (such as winter lighting protocols for mares^23^) should be balanced against potential disadvantages of foals being born during months when environmental conditions may be less favourable.

To the best of the authors’ knowledge, this is the first study to investigate associations between purchase price both as foal and yearling with race performance. Interestingly, the most expensive foals and yearlings ran fewer races than the less expensive ones. This seems counterintuitive because expensive horses might need to race more often to recover the investment; as suggested in a previous study, which observed a positive association between purchase price as yearling and the number of race starts.^11^ Our findings might therefore suggest that in the UK, expensive horses are purchased with a view to entering them in selected prestigious, higher value races, a strategy that may also reduce the risk of injury due to race overload.^24^

Despite the lower number of races run by expensive compared with less expensive horses, purchase price as foals and yearlings was positively associated with race performance by the end of the third year of life. The most expensive foals and yearlings earned more prize money and prize money per start as 3‐year olds than the less expensive ones. Furthermore, the more expensive a foal or a yearling was, the less likely it was for it not to win prize money at the end of its third year of life. Previous studies have reported an association between purchase price as yearling and race performance, measured either by Timeform ratings,^12^ or the number of starts, places and prize money earned.^11^

Prize money won during a racing career is an important return on the money invested to produce a TB racehorse.^1^ In our study, the proportion of horses that did not earn any prize money by the end of their second year of life (34%) was slightly lower than in an Australian study (39%).^10^ Overall, the prize money earned by the end of the third year of life was not higher than the purchase price paid as foal or yearling, with a low percentage (11%) of horses ‘earning’ enough money to at least cover their purchase price. Interestingly, although the median prize money earned by the end of the third year of life was similar for horses sold either as foals or yearling compared to horses never intended to be sold, the maximum prize money earned for the latter category was almost twice the maximum prize money earned by the former.

Several factors can influence an owner's decision to sell an individual, most notably whether their enterprise is commercial and reliant on sales income or whether they breed individuals to retain for racing. Health status is also important as horses are subject to veterinary examination and frequently extensive radiographic screening at sales.^25^ Management of these two subsets of the population is also likely to be different as those individuals presented for sale usually undertake several weeks of preparation where supplementary exercise and nutrition is provided which could also influence future performance.^26^, ^27^ It can be argued that if horses are not able to cover at least the cost of their purchase price, they will not be able to cover other associated production costs such as training fees, which could be even higher.^28^ Other studies have reported a very low proportion (5%) of horses covering the combined purchase price and estimated training costs^11^ or the estimated training fees by the end of the second (13%; 5%)^3^, ^10^ or third year of life (17%).^3^ Such findings, alongside results from our study, suggest low profitability in the horseracing industry—a concern highlighted in previous reports where up to two‐third of breeding operations were estimated to be unprofitable.^1^, ^2^ A thorough economic analysis is required to accurately estimate the profitability of breeding horses for racing to inform strategies to produce high‐quality horses more efficiently and improve the financial sustainability of breeding enterprises.

The associations found in the present study should not be interpreted as causal. Our analyses were limited by the information available in the dataset. Environmental factors that are likely to be observed after the yearling sales events such as trainer, choice of races entered, jockeys employed, track condition, year, season, class of race, distance and injury events,^22^, ^29^ may explain horse race performance better than sales data.^12^ However, a horse with good racing potential may be required for these environmental factors to have an impact. The explanatory variables included in the present study, even if they were measured very early in the horse's life, are likely to be proxy measures for its physical development or racing potential. Factors like DoB,^2^, ^30^ conformation and pedigree,^12^ sales category, sales event, stud fees, vendor category, purchaser country of origin and mare age,^2^ are known to influence yearling purchase price. Therefore, despite the limitations inherent to the use of existing, retrospective datasets for research purposes, our results provide useful information for the UK/IRE horseracing industry.

The scope for the present study was performance in flat races by the end of the third year of a horse's life. Including flat races results allowed us to explore race performance for 2‐year olds as an outcome. The study period was set to the end of the third year of horses’ lives to include complete information on both the 2014 and 2015 foal crops. At the time of data extraction, full 4‐year information was only available for the 2014 foal crop and only partial 4‐year information was available for the 2015 foal crop. The impact of these choices is likely to be very low provided that the effect (if any) of DoB on race performance is more evident in flat races than other types and decreases with the age of the horse.^4^ For instance, in horses older than 4 years, the effect of DoB on race performance seems to be stable and of smaller magnitude or tend to zero.^5^ A study analysing horses’ performance in different disciplines observed that the effect of DoB on performance was more evident in flat races than in other disciplines.^19^

In conclusion, horses born early during the foaling season had higher earnings by the end of their second and third years of life than horses born late. The closer to 1 January a foal is born, the more prize money and prize money per start it is likely to earn by the end of its second and third years of life. This better performance of early foals does not seem to be related to the number of races run. Purchase price as foals and yearlings was positively associated with race performance by the end of the third year of life. However, the most expensive horses sold as foals or yearlings ran fewer races than the less expensive ones. A thorough economic analysis to estimate the profitability of breeding horses for racing is needed given that a notable proportion of horses did not earn any money by the end of their third year of life and a low percentage of horses earned enough prize money to at least cover their purchase price.

## AUTHOR CONTRIBUTIONS

S*ubstantial contributions to conception and design of the study*: Juan Carlos Arango‐Sabogal, Kristien Verheyen, Rebecca Mouncey and Amanda M. de Mestre; *acquisition of data*; Kristien Verheyen; *analysis*: Juan Carlos Arango‐Sabogal; *interpretation of data*: Juan Carlos Arango‐Sabogal, Rebecca Mouncey, Amanda M. de Mestre and Kristien Verheyen. *Drafting the article*: Juan Carlos Arango‐Sabogal; *revising it critically for important intellectual content*: Rebecca Mouncey, Amanda M. de Mestre and Kristien Verheyen. *Final approval of the version to be published*: Juan Carlos Arango‐Sabogal, Rebecca Mouncey, Amanda M. de Mestre and Kristien Verheyen; *accountable for all aspects of the work*: Juan Carlos Arango‐Sabogal, Rebecca Mouncey, Amanda M. de Mestre, and Kristien Verheyen.

## CONFLICTS OF INTEREST

The authors declare that they have no conflicts of interest.

## ETHICS STATEMENT

Ethical approval was granted by the Royal Veterinary College Clinical Research Ethical Review Board (URN: 2018 1843‐2).

## Supporting information

Supporting Information

## Data Availability

Data were provided by Weatherbys and included publicly available and confidential data. Consent to use and store these data were subject to confidentiality agreements with both Weatherbys and the British Horseracing Authority. Thoroughbred racing data are publicly available.
