# Supplementary material for: Date of birth and purchase price as foals or yearlings are associated with Thoroughbred flat race performance in the United Kingdom and Ireland
Source: Vet Rec Open. 2022 Sep 23;9(1):e43. doi: 10.1002/vro2.43 (PMC9508327; doi:10.1002/vro2.43)
Supplement: Supplementary file 1 — Supporting Information [file VRO2-9-e43-s001.docx]

# **Supporting Information**

## Table S1 Purchase price categories (in Guineas) of the 2014-2015 UK/Ireland (IRE) Thoroughbred foal crop horses brought to sales events as foals and yearlings and raced at least once in UK/IRE flat races by the end of their 2nd and 3rd year of life

| **Price category as foal†** |  | **Price category as yearling†** | | | | | |
| --- | --- | --- | --- | --- | --- | --- | --- |
|  | Not sold as yearling | Went but not sold‡ | ≤ 6,666 | > 6,666 – 19,000 | > 19,000 – 48,000 | > 48,000 | Total |
| Not sold as foal | 2,770 | 377 | 625 | 772 | 1,013 | 1,112 | 6,669 |
| Went but not sold‡ | 90 | 50 | 89 | 103 | 101 | 66 | 499 |
| ≤ 5,167 | 63 | 20 | 122 | 49 | 18 | 1 | 273 |
| > 5,167 – 11,904 | 60 | 23 | 74 | 167 | 94 | 8 | 426 |
| > 11,904 – 25,396 | 50 | 31 | 35 | 158 | 263 | 73 | 610 |
| > 25,396 | 135 | 46 | 9 | 49 | 259 | 481 | 979 |
| Total | 3,168 | 547 | 954 | 1,298 | 1,748 | 1,741 | 9,456 |

† Purchase price categories were created using the quartiles of purchase price for horses sold.

‡ Horses that were brought to the sales event but were not sold.

## Table S2. Descriptive statistics of performance outcomes in UK/IRE flat races by the end of their 2^nd^ and 3^rd^ year of life of the horses from the 2014-2015 UK/Ireland (IRE) Thoroughbred foals crops that never went to the sales as foal or yearling

| **Age** | **Outcome** | **Median** | **Mean (SEM)** | **Standard deviation (SD)** | **Interquartile range (IQR)** | **Range** |
| --- | --- | --- | --- | --- | --- | --- |
| End of 2^nd^ year  (*n* = 1,723) | Number of races | 3 | 3.1 (0.05) | 2.1 | 1-4 | 1-17 |
|  | Prize money† | 626 | 6,022 (594.9) | 24,695 | 0-4,027 | 0-559,193 |
|  | Prize money† per start | 236 | 1,548 (124.1) | 5,152 | 0-1,270 | 0-93,198 |
|  |  |  |  |  |  |  |
| End of 3^rd^ year  (*n* = 2,770) | Number of races | 5 | 6.2 (0.08) | 4.2 | 3-8 | 1-31 |
|  | Prize money† | 3,072 | 16,154 (1382.4) | 72,754 | 241-9,909 | 0-1,367,500 |
|  | Prize money† per start | 498 | 2,208 (177.8) | 9,358 | 54-1,555 | 0-214,945 |

† Prize money in GBP (£).

## Table S3. Final negative binomial models to assess the association between purchase price as foal and yearling of the 2014-2015 UK/Ireland (IRE) Thoroughbred foal crops and the number of races by the end of the 3^rd^ year of life in UK/IRE flat racing

| **Age at sales** | |  | **β†** | **95% CI‡** | **Exp (β)** | **z** | ***P*** |
| --- | --- | --- | --- | --- | --- | --- | --- |
| ***Sold as foal (n = 2,787)*** | | |  |  |  |  |  |
|  | Purchase price as foal | Went/not sold§ | Ref. | - | - | *-* | - |
|  |  | ≤5,167 | 0.12 | 0.03; 0.21 | 1.13 | 2.6 | 0.010 |
|  |  | >5,167-11,904 | 0.13 | 0.05; 0.21 | 1.13 | 3.1 | 0.002 |
|  |  | >11,904-25,396 | 0.10 | 0.02; 0.17 | 1.10 | 2.5 | 0.012 |
|  |  | >25,396 | -0.01 | -0.08; 0.06 | 0.99 | -0.2 | 0.839 |
|  | Date of birth¶ |  | -0.004 | -0.01; -0.002 | 1.00 | -1.3 | 0.212 |
|  | Sex | Female | Ref. | - | - | *-* | - |
|  |  | Male | 0.13 | 0.08; 0.18 | 1.13 | 5.0 | <0.001 |
|  |  |  |  |  |  |  |  |
| ***Sold as yearling (n = 6,288)*** | | |  |  |  |  |  |
|  | Purchase price as yearling | Went/not sold§ | Ref. | - | - | *-* | - |
|  |  | ≤6,666 | 0.14 | 0.07; 0.21 | 1.15 | 4.1 | <0.001 |
|  |  | >6,666-19,000 | 0.17 | 0.11; 0.24 | 1.19 | 5.4 | <0.001 |
|  |  | >19,000-48,000 | 0.16 | 0.10; 0.22 | 1.18 | 5.2 | <0.001 |
|  |  | >48,000 | 0.02 | -0.05; 0.08 | 1.02 | 0.5 | 0.602 |
|  | Purchase price as foal | Not sold as foal | Ref. | - | - | *-* | - |
|  |  | Went/not sold§ | 0.02 | -0.05; 0.08 | 1.02 | 0.5 | 0.625 |
|  |  | ≤5,167 | 0.06 | -0.03; 0.15 | 1.06 | 1.4 | 0.162 |
|  |  | >5,167-11,904 | 0.10 | 0.03; 0.17 | 1.10 | 2.8 | 0.005 |
|  |  | >11,904-25,396 | 0.06 | 0.01; 0.12 | 1.06 | 2.2 | 0.026 |
|  |  | >25,396 | 0.05 | 0.00; 0.10 | 1.05 | 2.0 | 0.050 |
|  | Date of birth¶ |  | -0.003 | -0.01; 0.001 | 1.00 | -1.6 | 0.112 |
|  | Sex | Female | Ref. | - | - | *-* | - |
|  |  | Male | 0.08 | 0.05; 0.11 | 1.08 | 4.8 | <0.001 |

† β: regression coefficient.

‡ CI: confidence interval.

§ Horses that were brought to the sales event but were not sold.

¶ Measured as days after January 1^st^ of the corresponding year of birth and scaled to reflect the increase by every 7 days.

## Table S4. Final zero-inflated negative binomial models to assess the association between purchase price as foal (Model A) and yearling (Model B) of the 2014-2015 UK/Ireland (IRE) Thoroughbred foal crops and the prize money earned by the end of the 3rd year of life in UK/IRE flat racing

Estimates are adjusted by date of birth and sex (Model A) and date of birth, sex and purchase price as foal (Model B)

| **Model A (*n* = 2,787)** | | **Categories** | **β†** | **95% CI‡** | **Exp (β)** | **Z** | **P** |
| --- | --- | --- | --- | --- | --- | --- | --- |
| *Count part (negative binomial)* | | |  |  |  |  |  |
|  | Purchase price as foal | Went/not sold§ | Ref. | - | - | *-* | - |
|  |  | ≤5,167 | 0.26 | 0.04; 0.47 | 1.29 | 2.4 | 0.018 |
|  |  | >5,167-11,904 | 0.25 | 0.06; 0.43 | 1.28 | 2.7 | 0.008 |
|  |  | >11,904-25,396 | 0.34 | 0.17; 0.50 | 1.40 | 4.0 | <0.001 |
|  |  | >25,396 | 0.51 | 0.36; 0.66 | 1.66 | 6.6 | <0.001 |
|  | Date of birth¶ |  | -0.01 | -0.03; -0.001 | 0.99 | -2.6 | 0.030 |
|  | Sex | Female | Ref. | - | - | *-* | - |
|  |  | Male | 0.29 | 0.18; 0.40 | 1.34 | 5.2 | <0.001 |
|  |  |  |  |  |  |  |  |
| ***Binary part (logistic)*** | | |  |  |  |  |  |
|  | Purchase price as foal | Went/not sold§ | Ref. | - | - | *-* | - |
|  |  | ≤5,167 | 0.19 | -0.15; 0.53 | 1.21 | 1.1 | 0.269 |
|  |  | >5,167-11,904 | 0.05 | -0.25; 0.36 | 1.06 | 0.3 | 0.732 |
|  |  | >11,904-25,396 | -0.43 | -0.73; -0.12 | 0.65 | -2.8 | 0.006 |
|  |  | >25,396 | -0.73 | -1.02; -0.44 | 0.48 | -5.0 | <0.001 |
|  | Date of birth¶ |  | 0.03 | 0.01; 0.05 | 1.03 | 2.5 | 0.013 |
|  | Sex | Female | Ref. | - | - | *-* | - |
|  |  | Male | -0.42 | -0.63; -0.22 | 0.65 | -4.1 | <0.001 |
|  |  |  |  |  |  |  |  |
| **Model B (*n* = 6,288)** | | **Categories** | **β†** | **95% CI‡** | **Exp (β)** | **z** | ***P*** |
| *Count part (negative binomial)* | | |  |  |  |  |  |
|  | Purchase price as yearling | Went/not sold§ | Ref. | - | - | *-* | - |
|  |  | ≤6,666 | -0.40 | -0.56; -0.24 | 0.67 | -5.0 | <0.001 |
|  |  | >6,666-19,000 | -0.14 | -0.29; 0.001 | 0.87 | -2.0 | 0.051 |
|  |  | >19,000-48,000 | 0.17 | 0.04; 0.31 | 1.19 | 2.5 | 0.014 |
|  |  | >48,000 | 0.50 | 0.36; 0.63 | 1.64 | 7.0 | <0.001 |
|  | Purchase price as foal | Not sold | Ref. | - | - | *-* | - |
|  |  | Went/not sold§ | -0.17 | -0.31; -0.02 | 0.84 | -2.3 | 0.022 |
|  |  | ≤5,167 | 0.26 | 0.05; 0.47 | 1.30 | 2.5 | 0.014 |
|  |  | >5,167-11,904 | 0.16 | 0.002; 0.31 | 1.17 | 2.0 | 0.047 |
|  |  | >11,904-25,396 | -0.04 | -0.16; 0.09 | 0.96 | -0.6 | 0.539 |
|  |  | >25,396 | -0.05 | -0.16; 0.05 | 0.95 | -1.0 | 0.315 |
|  | Date of birth¶ |  | -0.01 | -0.02; -0.01 | 0.99 | -3.5 | <0.001 |
|  | Sex | Female | Ref. | - | - | *-* | - |
|  |  | Male | 0.26 | 0.18; 0.33 | 1.29 | 7.0 | <0.001 |
|  |  |  |  |  |  |  |  |
| ***Binary part (logistic)*** | | |  |  |  |  |  |
|  | Purchase price as yearling | Went/not sold§ | Ref. | - | - | *-* | - |
|  |  | ≤6,666 | 0.16 | -0.08; 0.40 | 1.17 | 1.3 | 0.199 |
|  |  | >6,666-19,000 | -0.33 | -0.57; -0.09 | 0.72 | -2.7 | 0.008 |
|  |  | >19,000-48,000 | -0.53 | -0.77; -0.30 | 0.59 | -4.4 | <0.001 |
|  |  | >48,000 | -0.91 | -1.16; -0.65 | 0.40 | -7.0 | <0.001 |
|  | Purchase price as foal | Not sold | Ref. | - | - | *-* | - |
|  |  | Went/not sold§ | 0.08 | -0.18; 0.34 | 1.08 | 0.6 | 0.569 |
|  |  | ≤5,167 | 0.17 | -0.16; 0.50 | 1.18 | 1.0 | 0.309 |
|  |  | >5,167-11,904 | 0.14 | -0.13; 0.41 | 1.15 | 1.0 | 0.317 |
|  |  | >11,904-25,396 | -0.06 | -0.31; 0.19 | 0.94 | -0.5 | 0.634 |
|  |  | >25,396 | -0.12 | -0.36; 0.11 | 0.88 | -1.0 | 0.304 |
|  | Date of birth¶ |  | 0.02 | 0.004; 0.03 | 1.02 | 2.4 | 0.016 |
|  | Sex | Female | Ref. | - | - | *-* | - |
|  |  | Male | -0.27 | -0.41; -0.14 | 0.76 | -3.9 | <0.001 |

† β: regression coefficient. ‡ CI: confidence interval. § Horses that were brought to the sales event but were not sold. ^d^ Measured as days after January 1 of the corresponding year of birth and scaled to reflect the increase by every 7 days.

## Table S5. Final zero-inflated negative binomial models to assess the association between purchase price as foal (Model A) and yearling (Model B) of the 2014-2015 UK/Ireland (IRE) Thoroughbred foal crops and the prize money per start earned by the end of the 3^rd^ year of life in UK/IRE flat racing

Estimates are adjusted by date of birth and sex (Model A) and date of birth, sex and purchase price as foal (Model B)

| **Model A: Sold as foal (*n* = 2,787)** | | **Categories** | **β†** | **95% CI‡** | **Exp (β)** | **z** | ***P*** |
| --- | --- | --- | --- | --- | --- | --- | --- |
| *Count part (negative binomial)* | | |  |  |  |  |  |
|  | Purchase price as foal | Went/not sold§ | Ref. | - | - | *-* | - |
|  |  | ≤5,167 | 0.11 | -0.09; 0.31 | 1.11 | 1.0 | 0.297 |
|  |  | >5,167-11,904 | 0.17 | -0.01; 0.34 | 1.18 | 1.9 | 0.061 |
|  |  | >11,904-25,396 | 0.38 | 0.23; 0.54 | 1.46 | 4.8 | <0.001 |
|  |  | >25,396 | 0.65 | 0.51; 0.79 | 1.92 | 8.9 | <0.001 |
|  | Date of birth¶ |  | -0.02 | -0.03; -0.004 | 0.98 | -2.6 | 0.011 |
|  | Sex | Female | Ref. | - | - | *-* | - |
|  |  | Male | 0.24 | 0.14; 0.35 | 1.28 | 4.6 | <0.001 |
| ***Binary part (logistic)*** | | |  |  |  |  |  |
|  | Purchase price as foal | Went/not sold§ | Ref. | - | - | *-* | - |
|  |  | ≤5,167 | 0.19 | -0.15; 0.54 | 1.21 | 1.1 | 0.269 |
|  |  | >5,167-11,904 | 0.06 | -0.26; 0.37 | 1.06 | 0.4 | 0.727 |
|  |  | >11,904-25,396 | -0.43 | -0.73; -0.12 | 0.65 | -2.7 | 0.007 |
|  |  | >25,396 | -0.74 | -1.03; -0.44 | 0.48 | -4.9 | <0.001 |
|  | Date of birth¶ |  | 0.03 | 0.01; 0.05 | 1.03 | 2.5 | 0.013 |
|  | Sex | Female | Ref. | - | - | *-* | - |
|  |  | Male | -0.43 | -0.63; -0.22 | 0.65 | -4.1 | <0.001 |
|  | |  |  |  |  |  |  |
| **Model B: Sold as yearling (*n* = 6,288)** | | **Categories** | **β†** | **95% CI‡** | **Exp (β)** | **z** | ***P*** |
| *Count part (negative binomial)* | | |  |  |  |  |  |
|  | Purchase price as yearling | Went/not sold§ | Ref. | - | - | *-* | - |
|  |  | ≤6,666 | -0.53 | -0.68; -0.38 | 0.59 | -7.0 | <0.001 |
|  |  | >6,666-19,000 | -0.27 | -0.41; -0.13 | 0.76 | -3.9 | <0.001 |
|  |  | >19,000-48,000 | 0.08 | -0.05; 0.21 | 1.08 | 1.2 | 0.223 |
|  |  | >48,000 | 0.54 | 0.41; 0.67 | 1.71 | 8.2 | <0.001 |
|  | Purchase price as foal | Not sold | Ref. | - | - | *-* | - |
|  |  | Went/not sold§ | -0.23 | -0.37; -0.09 | 0.79 | -3.3 | 0.001 |
|  |  | ≤5,167 | 0.20 | 0.003; 0.40 | 1.22 | 2.0 | 0.047 |
|  |  | >5,167-11,904 | 0.07 | -0.08; 0.21 | 1.07 | 0.9 | 0.374 |
|  |  | >11,904-25,396 | -0.02 | -0.13; 0.10 | 0.98 | -0.3 | 0.769 |
|  |  | >25,396 | -0.03 | -0.12; 0.07 | 0.98 | -0.5 | 0.613 |
|  | Date of birth¶ |  | -0.01 | -0.01; -0.001 | 0.99 | -2.1 | 0.036 |
|  | Sex | Female | Ref. | - | - | *-* | - |
|  |  | Male | 0.19 | 0.12; 0.26 | 1.21 | 5.6 | <0.001 |
| ***Binary part (logistic)*** | | |  |  |  |  |  |
|  | Purchase price as yearling | Went/not sold§ | Ref. | - | - | *-* | - |
|  |  | ≤6,666 | 0.16 | -0.09; 0.40 | 1.17 | 1.2 | 0.216 |
|  |  | >6,666-19,000 | -0.34 | -0.58; -0.09 | 0.71 | -2.7 | 0.007 |
|  |  | >19,000-48,000 | -0.54 | -0.78; -0.30 | 0.58 | -4.4 | <0.001 |
|  |  | >48,000 | -0.91 | -1.17; -0.65 | 0.40 | -6.9 | <0.001 |
|  | Purchase price as foal | Not sold | Ref. | - | - | *-* | - |
|  |  | Went/not sold§ | 0.07 | -0.19; 0.34 | 1.08 | 0.6 | 0.585 |
|  |  | ≤5,167 | 0.17 | -0.16; 0.50 | 1.19 | 1.0 | 0.304 |
|  |  | >5,167-11,904 | 0.14 | -0.13; 0.42 | 1.15 | 1.0 | 0.316 |
|  |  | >11,904-25,396 | -0.06 | -0.32; 0.19 | 0.94 | -0.5 | 0.631 |
|  |  | >25,396 | -0.13 | -0.37; 0.11 | 0.88 | -1.0 | 0.303 |
|  | Date of birth¶ |  | 0.02 | 0.004; 0.03 | 1.02 | 2.4 | 0.016 |
|  | Sex | Female | Ref. | - | - | *-* | - |
|  |  | Male | -0.28 | -0.41; -0.14 | 0.76 | -3.9 | <0.001 |

† β: regression coefficient. ‡ CI: confidence interval. § Horses that were brought to the sales event but were not sold. ¶ Measured as days after January 1 of the corresponding year of birth and scaled to reflect the increase by every 7 days.
